# Effect of *Bacillus subtilis* Strains on Intestinal Barrier Function and Inflammatory Response

**DOI:** 10.3389/fimmu.2019.00564

**Published:** 2019-03-29

**Authors:** Lamya Rhayat, Marc Maresca, Cendrine Nicoletti, Josette Perrier, Karoline Sidelmann Brinch, Sonja Christian, Estelle Devillard, Erik Eckhardt

**Affiliations:** ^1^Centre d'Excellence en Recherche Nutritionelle, Adisseo SAS, Malicorne, France; ^2^Aix Marseille Univ., CNRS, Centrale Marseille, iSm2, Marseille, France; ^3^Novozymes A/S, Bagsvaerd, Denmark

**Keywords:** *Bacillus subtilis*, probiotics, digestive health, intestinal epithelium, inflammation, IL-8, NF-κB, tight junctions

## Abstract

Strong tight junctions and curtailed inflammatory responses under stressful conditions are key for optimal digestive health. *Bacillus*-based probiotics are increasingly being used to maintain broilers' health, but their mode of action is often not well-defined. In the present study we used Caco-2 cells as a model for intestinal epithelia and assessed the effect of three *Bacillus*-based probiotics on intestinal barrier function and intestinal inflammation. Experimental results showed that one of the three tested strains, Bs 29784, significantly reinforced intestinal barrier integrity under basal conditions through an up-regulation of the expression of tight junction's proteins, whereas the others had no or detrimental effects. When Caco-2 cells were pre-treated with *Bacillus subtilis* strains, the subsequent IL-8 release to various pro-inflammatory signals (IL-1β, deoxynivalenol, or flagellin) was blunted compared to cells that had not been pretreated, but to a different extent depending on the strain of *Bacillus* used. Bs 29784, was able to significantly decrease IL-8 production in all stressed conditions tested. Mechanistically, Bs 29784 appeared to limit nuclear translocation of NF-κB during IL-1β exposure by preventing IκB degradation. The effects of Bs 29784 were observed independently with supernatant and cells but in a lesser extent than with the combination, indicating that they can thus likely be attributed to both secreted metabolites and cell-associated compounds. Moreover, under inflammatory conditions, Bs 29784 significantly reduced the upregulation of iNOS protein levels further underlining its intestinal anti-inflammatory potential. Our data show that *Bacillus*-based probiotics may indeed improve digestive health by strengthening intestinal barrier and limiting inflammatory responses and that these properties are strain-dependent.

## Introduction

The intestine presents a large interface with the outside world, which enables efficient absorption of nutrients but also contains large numbers of micro-organisms, including opportunistic pathogens (1). The intestine is also regularly exposed to viruses, toxins, antigenic nutrients, dietary and endogenous surfactants, and other potentially harmful substances that may enter via the diet. A protective layer of mucus, secreted antibodies, and antimicrobial factors greatly reduces exposure of intestinal epithelial cells (IEC) to these threats (2, 3) and their translocation across the intestinal epithelium is further limited by inter-cellular tight junctions between IEC (4). Temporary weakening of the intestinal barrier may contribute to the development of intestinal inflammatory disorders such as inflammatory bowel disease in humans (5) or necrotic enteritis in broiler chicken (6, 7). Since inflammatory cytokines impair gut barrier integrity (8), and intestinal barrier disruption may also promote inflammation, it is important for IEC to limit inflammatory responses and to prevent intestinal barrier disruption.

Interestingly, some pro-inflammatory substances, of which there are many in the intestine, may actually strengthen the intestinal barrier (9) and stimulate expression of anti-microbial factors (3), immunoglobulin-A excreting polymeric Ig receptors (10), or mucin production by Goblet cells (11). Microbial Toll Like Receptor 2 (TLR2) agonists promote tight junction assembly and gene expression (4). Nevertheless, exposure of IEC to microbial compounds such as flagellin, toxins, or cytokines from adjacent cells, can stimulate secretion by IEC of pro-inflammatory cytokines such as interleukin 8 (IL-8) (12–14) and weaken tight junctions, thus potentially initiating an inflammatory response.

Many different micro-organisms reside in the intestine or arrive there via the diet. Some species and strains are known to exert pro-inflammatory effects on the intestinal barrier whereas others are anti-inflammatory such as *Bacillus* (7, 15–20). Due to their anti-inflammatory (and other) properties, many have been introduced as probiotics which, by definition, are live microorganisms that, when ingested, confer a health benefit to the host (21). In animal feed applications, the use of some *Bacillus* species (*B. subtilis, B. amyloliquefaciens, B. licheniformis*, or *B. pumilus*, for example) as probiotics became popular due to their spore-forming properties which makes them easy to handle (16) and to their ability to replace antibiotics as growth promoters (22). Among the different species of *Bacillus* used as probiotics in poultry, the *B. subtilis* strain 29784 (Bs 29784) has shown *in vivo* benefits in broilers, such as improvement of performance parameters under different rearing conditions (23).

In order to evaluate the direct anti-inflammatory potential of *B. subtilis* strains, we used an *in vitro* cell model. Human colonic epithelial cell lines, such as Caco-2 cells, are largely used as a model mimicking the intestinal barrier and to investigate the mucosal host response. When grown on permeable supports, Caco-2 cells polarize and form a monolayer with functionally and morphologically different apical and basolateral surfaces with solid intercellular tight junctions, thus resembling to enterocytes lining the small intestine. Inflammatory responses in Caco-2 cells can be elicited with a variety of relevant stimuli such as the potent activator of Toll-like Receptor 5, flagellin (24), the mycotoxin deoxynivalenol (DON) (14, 25), or cytokines such as interleukin 1 beta (IL-1β). IL-1β is also known to impair tight junctions in Caco-2 cells (26) and stimulates release of pro-inflammatory cytokines IL-6 (27) and IL-8 (12, 28). Since mammals and birds share common mechanisms of digestive health and (mucosal) immunity, express similar chemo- and cytokines, and even have similar microbial communities, it is reasonable to assume that Caco-2 cells, despite their human origin and with all limitations that this implies, may serve as a model for avian IEC until avian models become available. Thus, we used Caco-2 cells as a model for the intestinal epithelium to study the effect of the three *B. subtilis* strains (Bs 29784, Bs A and Bs B) on the barrier integrity and their ability to limit inflammatory responses upon stimulation with various pro-inflammatory substances.

NF-κB is well-known to play a central role in regulating gene expression in a wide variety of cellular responses, including immune response and inflammation. In regard to the conditions inducing an inflammatory response used in this study, we targeted the canonical NF-κB signaling pathway in order to shed some light on Bs 29784 mode of action (29, 30). In addition to pro-inflammatory cytokines and chemokines, such as IL-8, NF-κB directly induces the upregulation of inducible Nitric Oxide synthase (iNOS) also involved in intestinal inflammation (31, 32). Though important for the destruction of bacteria and viruses, NO produced by iNOS may have undesirable effects and needs to be controlled (33, 34).

The objective of the present study was to (1) assess *in vitro* the direct effect of Bs 29784 in induced inflammation conditions, in comparison with two other commercially available *B. subtilis* strains (Bs A and Bs B) used as probiotics in animal feed, and to (2) investigate how Bs 29874 could intervene in the NF-κB signaling pathway to modulate the inflammatory response.

## Materials and Methods

### Bacterial Strains and Cell Cultures

The bacterial strains used in the study (Bs 29784, Bs A and Bs B) were all commercially available *Bacillus subtilis* strains used as probiotics in animal feed. For experimental purposes, bacteria were grown from single colonies on Luria–Bertani (LB) plates in LB broth at 37°C overnight with shaking (200 rpm). The next day, overnight cultures were diluted 1:100 in LB broth and left to grow at 37°C under agitation (200 rpm) with regular measurement of the optical density (OD) at 600 nm until OD_600nm_ reached 0.2–0.3. Bacteria were then diluted in DMEM without FCS and without antibiotic to obtain the appropriate bacterial density (10^E5^, 10^E6^, or 10^E7^ bacteria/ml).

Caco-2 cells (obtained from ATCC) were maintained in Dulbecco's Modified Essential Medium (DMEM) supplemented with 10% fetal calf serum (FCS), 1% L-glutamine, and 1% antibiotics (all from Invitrogen) in a 5% CO_2_ incubator, at 37°C. Prior to each experiment, Caco-2 cells were seeded at a density of 250,000 cells per cm^2^ onto Greiner filter inserts (“ThinCert”; 1 cm^2^; pore size 0.4 μm) and left to differentiate with medium changed every 2 days. After 10–14 days of differentiation, epithelial tightness and cellular differentiation were evaluated by measurement of the transepithelial electrical resistance (TEER) using a Volt/Ohm meter (Millipore). Only tight inserts with a TEER superior or equal to 150 Ω.cm^−2^ were used experimentally.

### Cytokine Release and TEER Measurements

Bacteria were added in the apical compartment of the Caco-2 cells inserts. Basolateral compartment of the inserts were filled with DMEM without FCS or antibiotics. Inserts were then incubated for 16 h at 37°C in a 5% CO_2_ incubator when pro-inflammatory signals were applied. Deoxynivalenol (DON, 10 μM final concentration, obtained from Romer lab) was added to the apical compartment, while human recombinant IL-1β (20 ng/ml final concentration, obtained from Peprotech), or flagellin (1 μg/ml final concentration, extracted from *Salmonella typhimurium*, obtained from Invivogen) were added basolaterally to Caco-2. Epigallocatechin gallate (EGCG, 10 μM final concentration, obtained from Sigma Aldrich) was added to the apical compartment and used as positive anti-inflammatory control. After 6 h at 37°C, basolateral media were collected and stored at −80°C before IL-8 cytokine measurement by ELISA (BD Biosciences) was performed. TEER was measured using a Volt/Ohm meter (Millipore).

### Analysis of NF-κB Signaling Pathway Activation

The effect of bacteria on NF-κB signaling pathway was performed as previously described (35, 36). Briefly, Caco-2 cells seeded onto inserts were incubated with bacteria for 16 h at 37°C. At the end of the incubation, cells exposed or not to bacteria were stimulated basolaterally with human recombinant IL-1β (20 ng/ml final concentration, from Peprotech) for 0, 15, 30, or 60 min at 37°C. Nuclear and cytosolic fractions were then extracted. Briefly, cells were washed 3 times with cold phosphate buffer saline (PBS), scraped into cold PBS before resuspension in a buffer containing 10 mM Hepes (pH 7.8, 10 mM KCl, 2 mM MgCl_2_, 0.1 mM EDTA, 1 mM DTT, 1.25 mM NaF, 1 mM NaVO4, 1 mM PMSF) and incubated on ice for 15 min. Nonidet P-40 (0.5% final concentration) was then added. The obtained mixture was vortexed for 15 s prior to centrifugation (13,000 g, 4°C, 30 s) to pellet nuclei. The soluble supernatants corresponding to cytosolic fractions were resuspended in Laemmli sample buffer. The pelleted nuclei were resuspended in a buffer containing 50 mM Hepes (pH 7.8, 50 mM KCl, 300 mM NaCl, 10% glycerol, 0.1 mM EDTA, 1 mM DTT, 0.2 mM NaF, 0.2 mM NaVO4, 1 mM PMSF) and agitated for 20 min at 4°C (briefly vortexed every 5 min). The samples were finally centrifuged (13,000 g, 4°C, 5 min) and supernatants containing nuclear proteins were resuspended in Laemmli sample buffer. Samples were boiled for 10 min and analyzed by Western blotting. To this end, nuclear and cytosolic samples were separated on 4–12% precast SDS-PAGE gels (ThermoFisher) before transfer onto a nitrocellulose membrane. The membrane was then incubated for 1 h at room temperature in PBS containing 2% bovine serum albumin, washed with PBS containing Tween-20 at 0.05%, and incubated overnight at 4°C with the appropriate primary antibody diluted in PBS supplemented with 2% bovine serum albumin as per manufacturer's suggestions (i.e., 1:1,000 dilution). Rabbit monoclonal antibody against human NF-κB/p65 (C22B4) and mouse monoclonal antibody against IκBα (L35A5) were obtained from Cell Signaling Technology. Membranes were washed 3 times with PBS and incubated for 1 h with alkaline phosphatase-conjugated goat-anti-rabbit (for NF-κB/p65) or anti-mouse (for IκBα) immunoglobulin G secondary antibodies (Sigma Aldrich). Membranes were finally washed extensively with PBS Tween-20 and developed with alkaline phosphatase substrate (NBT/BCIP, Pierce). Band densities were measured using Image J software.

### Incubation of Caco-2 Cells With Secreted and Cell-Associated Bacterial Factors

To evaluate the impact of Bs 29784 secreted factors (SF) on the immune response, Caco-2 cells seeded onto 1 cm^2^ inserts (12-well inserts; pore size 0.4 μm) were exposed to live bacteria (initial bacterial density of 10^7^ bacteria/ml) on top of a 0.3 cm^2^ insert (24-well inserts, pore size 0.4 μm). This insert was positioned in the apical compartment of the Caco-2 insert, thus preventing direct contact between bacteria and Caco-2 cells. After 16 h at 37°C, Caco-2 cells were then stimulated with IL-1β or with a mixture of human recombinant pro-inflammatory cytokines called Cytomix (IFN-γ at 2,000 U/ml final concentration, TNF-α at 100 ng/ml final concentration, and IL-1β at 20 ng/ml final concentration, all purchased from PeproTech). Concerning the cell-associated factors (CAF), Caco-2 cells seeded onto 1 cm^2^ inserts were exposed to paraformaldehyde (PFA)-killed bacteria. Briefly, these bacteria were grown in LB broth to reach 0.6 OD_600nm_, washed with PBS, and then killed by 30 min exposure to PFA (4% final concentration). Bacteria were then washed three times with PBS and resuspended in DMEM to reach a bacterial density equivalent to 10^E9^ bacteria/ml. Killing of bacteria was confirmed by the absence of colonies on LB agar plates. Caco-2 cells were finally exposed for 16 h at 37°C to killed bacteria before stimulation with IL-1β or Cytomix.

### iNOS Western Blotting

The effect of bacteria on the induction of iNOS by pro-inflammatory cytokines was also evaluated as previously described (33, 34). Briefly, Caco-2 cells seeded onto inserts were incubated with bacteria for 16 h at 37°C. At the end of the incubation, Caco-2 cells were basolaterally exposed to Cytomix (IFN-γ at 2,000 U/ml final concentration, TNF-α at 100 ng/ml final concentration, and IL-1β at 20 ng/ml final concentration, all purchased from PeproTech). iNOS protein levels were then measured as previously described (34, 37). After 24 h of incubation, cells were washed with cold PBS and solubilized on ice with 100 μl of lysis buffer (1% Triton X-100 in cold PBS) containing a protease inhibitor cocktail (from Sigma Aldrich). Protein concentrations were determined with the Bradford Reagent (Sigma Aldrich) following the manufacturer's instructions. Twenty-five microliter of 5X Laemmli sample buffer were added and samples boiled for 10 min. Samples containing 50 μg of protein were separated on 4–12% precast SDS-PAGE gels (ThermoFisher) before transfer to a nitrocellulose membrane. The membrane was incubated for 1 h at room temperature in PBS containing 2% bovine serum albumin, washed with PBS, and incubated for 1 h with the appropriate primary antibody diluted in PBS as per manufacturer's suggestions (i.e., 1:100 dilution). Rabbit polyclonal antibodies against human iNOS (sc-651) and actin (A-2066) were obtained from Santa Cruz and Sigma Aldrich, respectively. Membranes were then washed 3 times with PBS containing Tween-20 at 0.05 % and incubated for 1 h with alkaline phosphatase-conjugated goat anti-rabbit immunoglobulin G secondary antibody (Sigma Aldrich). Membranes were finally washed at least three times with PBS Tween-20 and revealed with alkaline phosphatase substrate (NBT/BCIP, Pierce). Band densities were measured using Image J software and iNOS levels being normalized to actin staining.

### Tight Junction Proteins Western Blotting

The effect of bacteria on the expression of tight junction proteins was evaluated using Western-blot. Briefly, Caco-2 cells seeded onto inserts were left untreated (control) or were treated apically with Bs 29784 (initial bacterial density of 10^7^ bacteria/ml) or with DON at 100 μM for 16 h at 37°C. At the end of the incubation, Caco-2 cells were washed three times with cold PBS and solubilized on ice with 100 μl of lysis buffer (1% Triton X-100 in cold PBS) containing a protease inhibitor cocktail (from Sigma Aldrich). Protein concentrations were determined with the Bradford Reagent (Sigma Aldrich) following the manufacturer's instructions. Twenty-five microliter of 5X Laemmli sample buffer were added and samples boiled for 10 min. Samples containing 50 μg of protein were separated on 4–12% precast SDS-PAGE gels (ThermoFisher) before transfer to a nitrocellulose membrane. The membrane was incubated for 1 h at room temperature in PBS containing 2% bovine serum albumin, washed with PBS, and then incubated with the appropriate primary antibody diluted in PBS as per manufacturer's suggestions (i.e., 1:100 dilution). Antibodies used were rabbit polyclonal antibodies against ZO-1 (ThermoFisher, reference 40–2200) or Occludin (ThermoFisher, reference 40–6100) and mice monoclonal antibody against Claudin-1 (ThermoFisher, reference 37–4900). After 1 h, membranes were washed 3 times with PBS containing Tween-20 at 0.05% and incubated for 1 h with alkaline phosphatase-conjugated goat anti-rabbit immunoglobulin G secondary antibody (Sigma Aldrich). Membranes were finally washed three times with PBS Tween-20 and revealed with alkaline phosphatase substrate (NBT/BCIP, Pierce). Band densities were measured using Image J software and levels of expression of tight junction proteins were normalized using actin staining.

### Statistical Analysis

Raw data from IL-8 measurement assays were subjected to ANOVA procedure of XLSTAT, with treatment as fixed effects. Results are reported as least square means with bars representing the standard error of the mean (SEM). LS means were assumed statistically different at *P* ≤ 0.05.

A two-tailed *t*-test was used to compare groups in Western blotting assays. Results are reported as least square means with bars representing the standard deviation (SD). LS means were assumed statistically different at *P* ≤ 0.05.

## Results

### Effects on TEER Are Strain Specific

**Figure 1:**
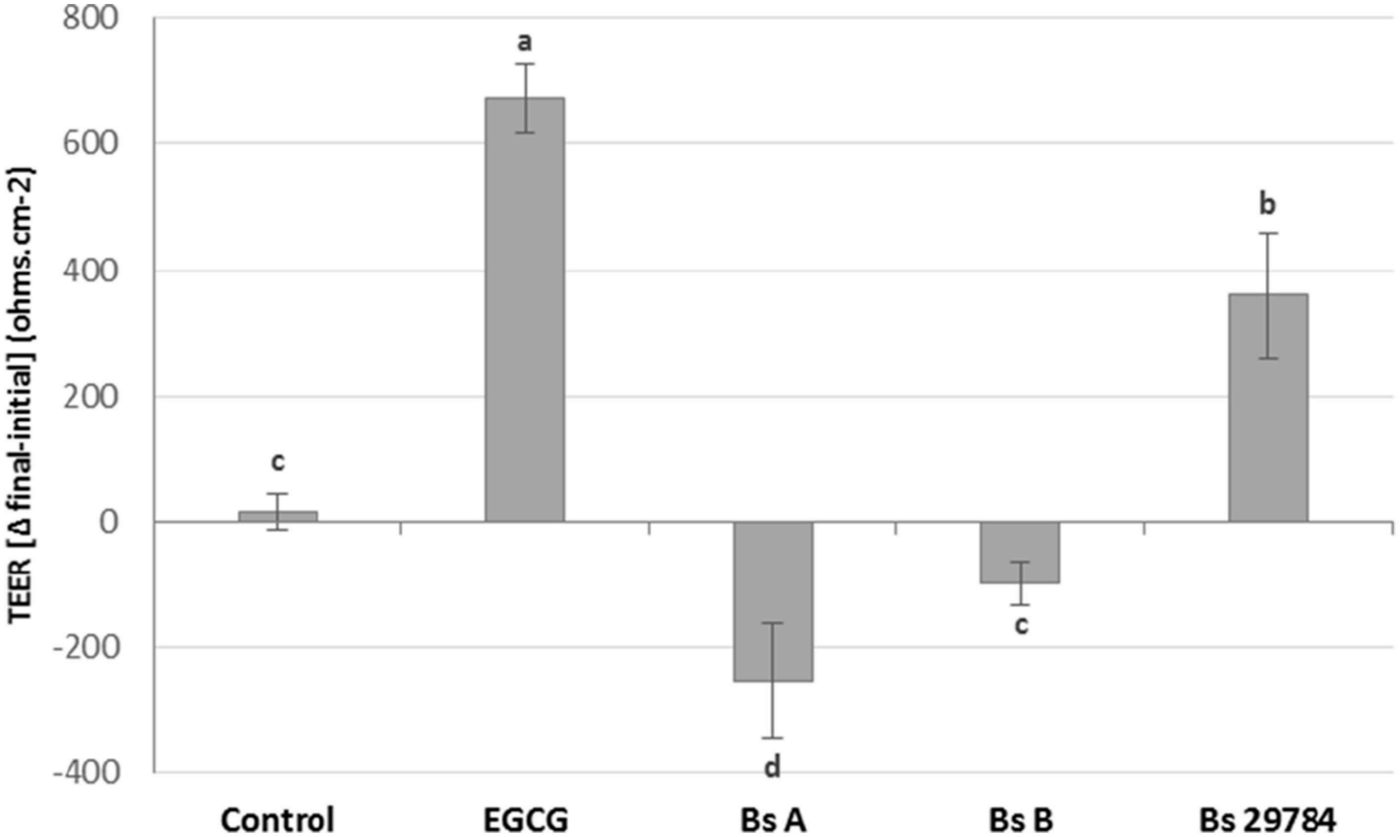
Effect of different *Bacillus* strains on TEER of differentiated Caco-2 cells. These cells had been exposed for 16 h on their apical surface with the indicated strains (10^7^ CFU/ml) or with EGCG (10 μM) prior to TEER measurement. Results are expressed as difference between final vs. initial TEER. Labeled means without a common letter differ, *p* < 0.05.

**Figure 2:**
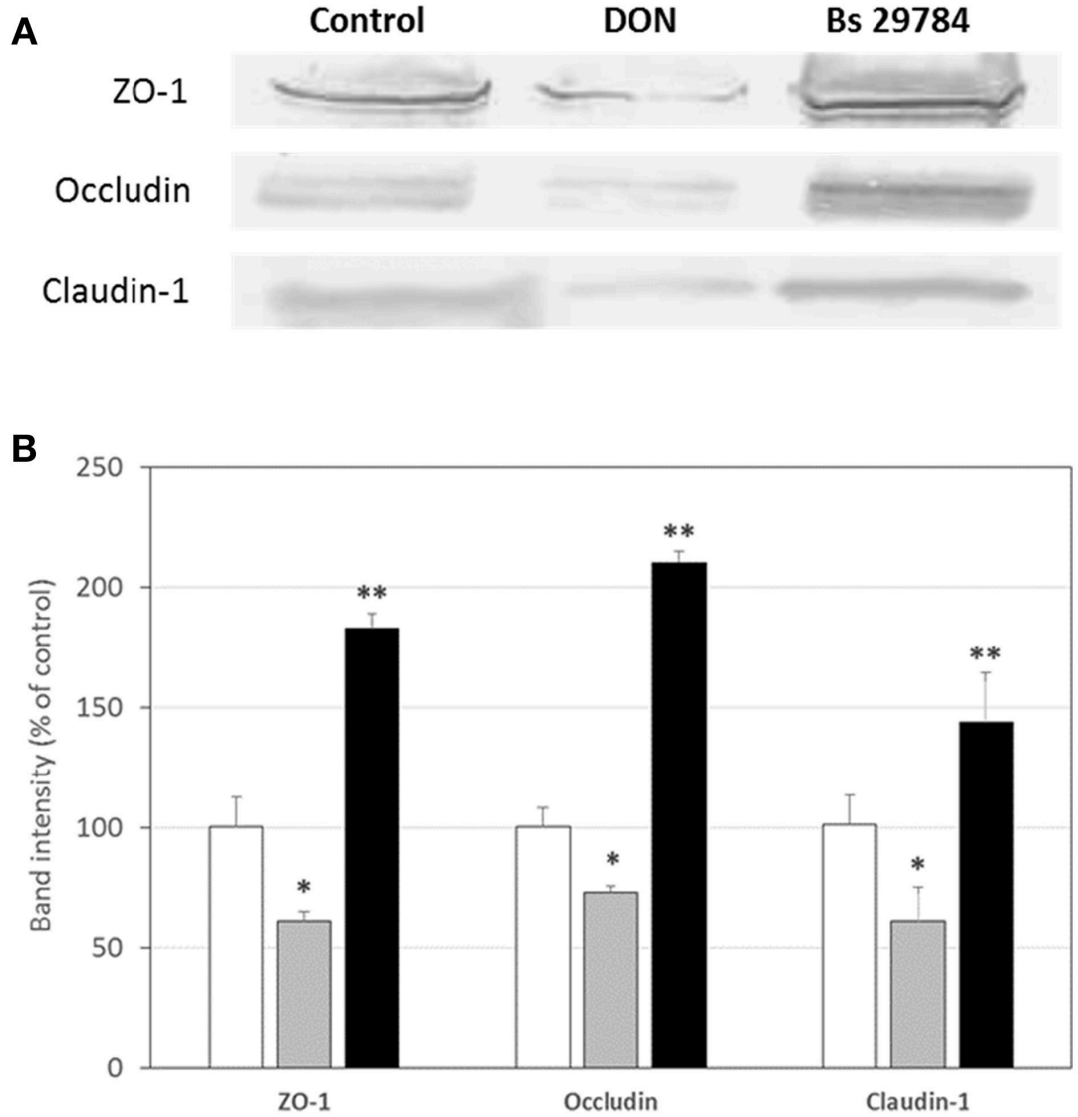
Bs 29784 increases the expression of tight junction's proteins. Caco-2 cells were left untreated (white columns) or were treated apically for 16 h with DON at 100 μM (gray columns) or Bs 29784 at an initial bacterial density of 10^7^ bacteria/ml (black columns). ZO-1, occludin, and claudin-1 proteins were visualized by Western-blot **(A)**. Band densities were measured using Image J software and subjected to statistical analysis **(B)**. ^*^ and ^**^Significant differences between the groups, *p* < 0.05 and *p* < 0.01, respectively.

Growth of the three *B. subtilis* strains in culture media being similar, we used the same concentration of each, allowing us a direct comparison between the strains (results not shown). Preliminary tests had shown that the three strains had a dose-dependent effect on TEER, with a maximal effect at 10^7^ CFU/ml. Therefore, this dose was chosen for future experiments. When Caco-2 cells were apically exposed for 16 h with the various strains, subsequent TEER measurements showed that the three *B. subtilis* strains behaved differently (Figure 1). In this experiment, we used Epigallo Catechin Gallate (EGCG) -an established anti-inflammatory compound (38) as positive control. As expected, EGCG significantly (*p* < 0.001) increased the TEER (*p* < 0.001), thus validating the experiment. Whereas, Bs 29784 caused a significant increase in TEER of 50.7% (*p* < 0.001) compared to the unstimulated control, Bs B had no significant effect (*p* = 0.107), while Bs A caused a significant decrease in TEER of about 40% (*p* < 0.01). The increase in TEER caused by Bs 29784 was correlated to an increase in the expression of the main proteins involved in the formation of tight junctions (Figure 2). Thus, whereas exposure to DON is associated to a decrease in the expression of ZO-1, occludin and claudin-1 (i.e., 40.3, 27, and 39.1% decrease, respectively) Bs 29784 caused an increase in their expression (i.e., 82.9, 110.2, and 43.9% increase in ZO-1, occludin and claudin-1 expression, respectively).

### Immunomodulatory Properties Are Strain Specific

**Figure 3:**
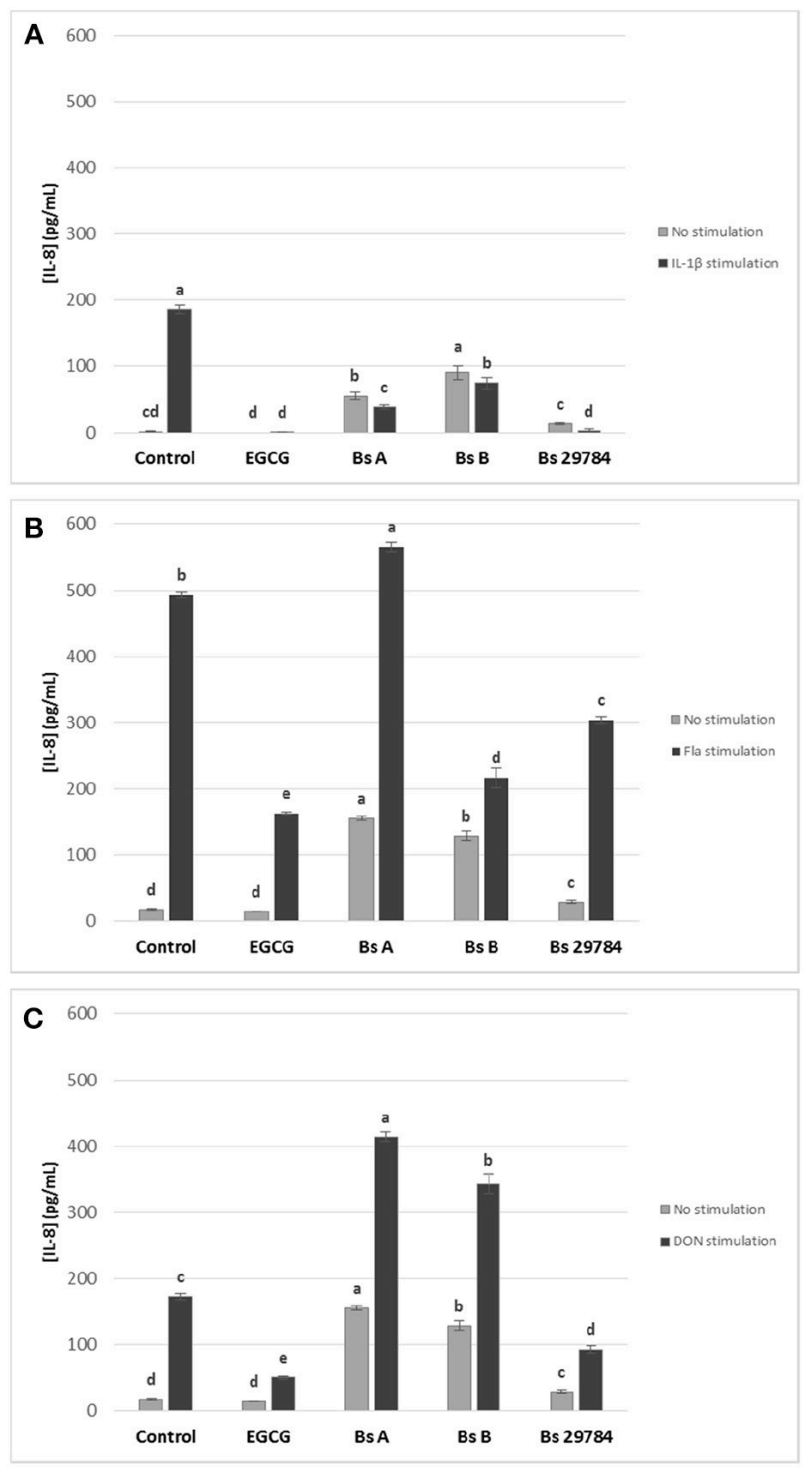
Effect of apical application for 16 h of different *B. subtilis* strains on IL-8 release of Caco-2 cells with or without subsequent 6 h basolateral stimulation with **(A)** IL-1β (20 ng/ml) or **(B)** flagellin (1 μg/ml), or **(C)** apical stimulation with DON (10 μM). EGCG was used as positive control. Labeled means without a common letter differ within each condition, *p* < 0.05.

We next evaluated the effect of the three *B. subtilis* strains on intestinal inflammation by measuring basolateral release of IL-8 from Caco-2 cells exposed apically to *Bacillus* for 16 h prior to stimulation for 6 h with various pro-inflammatory compounds: IL1-β, Deoxynivalenol (DON) and flagellin (Fla) (Figure 3). EGCG was here again used as an anti-inflammatory control. IL-8 release by Caco-2 cells was increased by all three stimuli. EGCG pretreatment decreased IL-8 release induced in all stressed conditions. Not unexpectedly, all *B. subtilis* strains by themselves stimulated IL-8 production. However, all tested strains were able to attenuate IL-1β-induced IL-8 production by 61.3, 65.3, and 74.8% with Bs A, Bs B, and Bs 29784, respectively, compared to Caco-2 cells with no pre-treatment (Figure 3A). Nevertheless, the strongest anti-inflammatory effect in this condition was obtained with Bs 29784 when compared to the two other *B. subtilis* strains. In flagellin-mediated inflammation, we observed that Bs A caused a 14.9% increase (*p* < 0.01) in IL-8 release, whereas Bs B and Bs 29784 decreased it by 56.1% (*p* < 0.0001) and 38.2% (< 0.0001), respectively (Figure 3B). Interestingly, Bs A and Bs B actually potentiated the effect of DON on IL-8 production (+139.0 and +97.7%, respectively), whereas Bs 29784 reduced IL-8 production by 46.4% (*p* < 0.0001) compared to DON-treated control (Figure 3C). Together, these results further illustrate the differences between *Bacillus* strains. Since Bs 29784 showed a significant decrease in IL-8 release in all inflammatory conditions, we subsequently investigated this strain in more detail.

### Protective Effects of Bs 29784 Involve Inhibition of the NF-κB Nuclear Translocation in IL-1β Stressed Conditions

**Figure 4:**
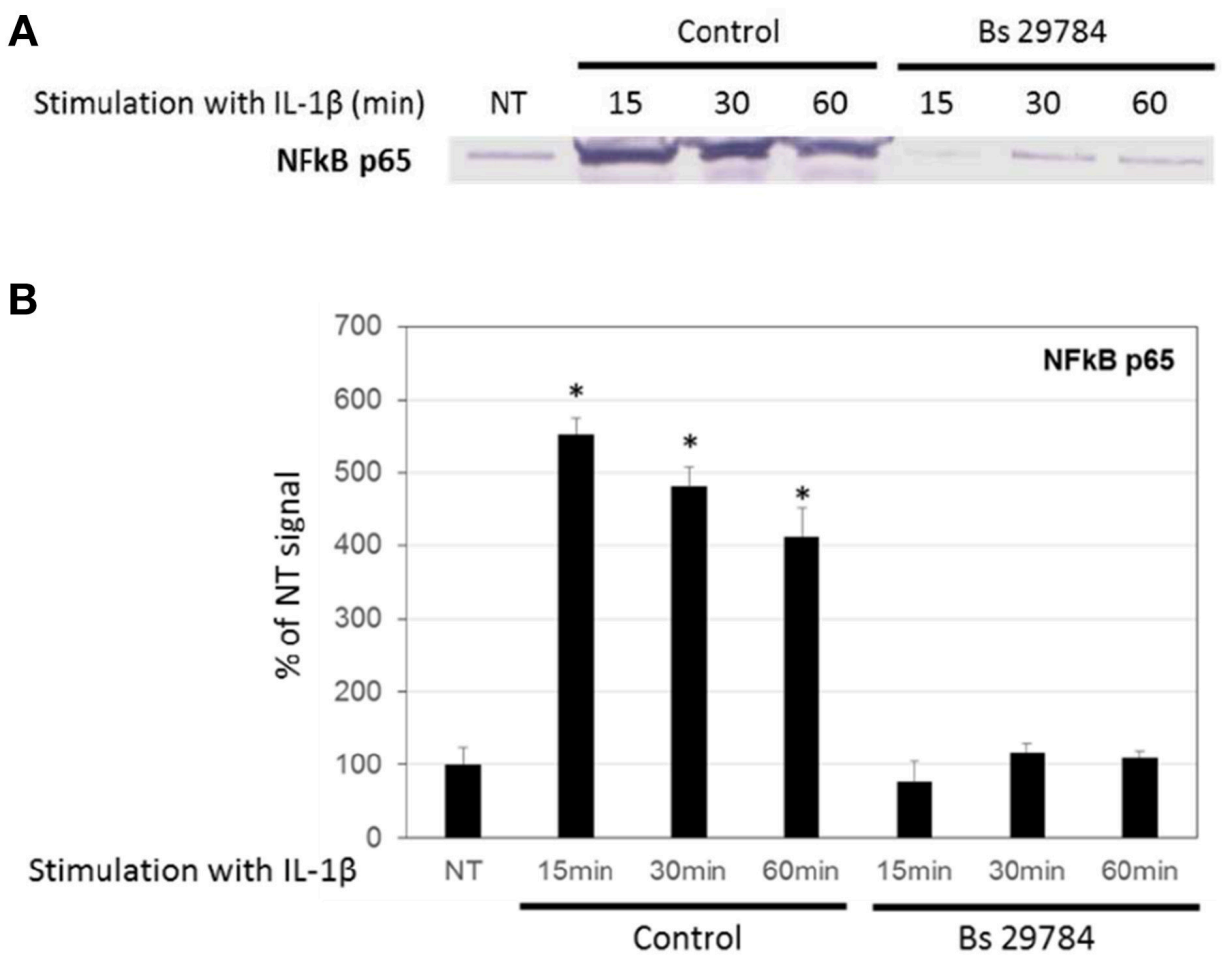
Inhibition of nuclear translocation of NF-κB in Caco-2 cells with Bs 29784. Caco-2 cells were pretreated apically with Bs 29784 for 16 h prior to basolateral stimulation with IL-1β (20 ng/ml) for 0, 15, 30, or 60 min. Nuclear levels of NF-κB were visualized by Western-blot **(A)**. Band densities were measured using Image J software and subjected to statistical analysis **(B)**. ^*^Significant differences between the groups, *p* < 0.05.

A key step in many inflammatory processes is the translocation of transcription factor NF-κB from the cytosol into the nucleus, where it drives the expression of pro-inflammatory cytokines including IL-8. Indeed, we observed an increase in the amount of NF-κB/p65 in the nucleus of Caco-2 cells stimulated with IL-1β (Figures 4A,B; 5.5-, 4.8-, and 4.1-fold increase compared to initial nuclear signal after 15, 30 and 60 min, respectively). However, when the Caco-2 cells had been pre-incubated for 16 h with Bs 29784, IL-1β-induced nuclear translocation of NF-κB/p65 was prevented (0.76-, 1.17-, and 1.09-fold increase compared to initial nuclear signal after 15, 30, and 60 min, respectively).

**Figure 5:**
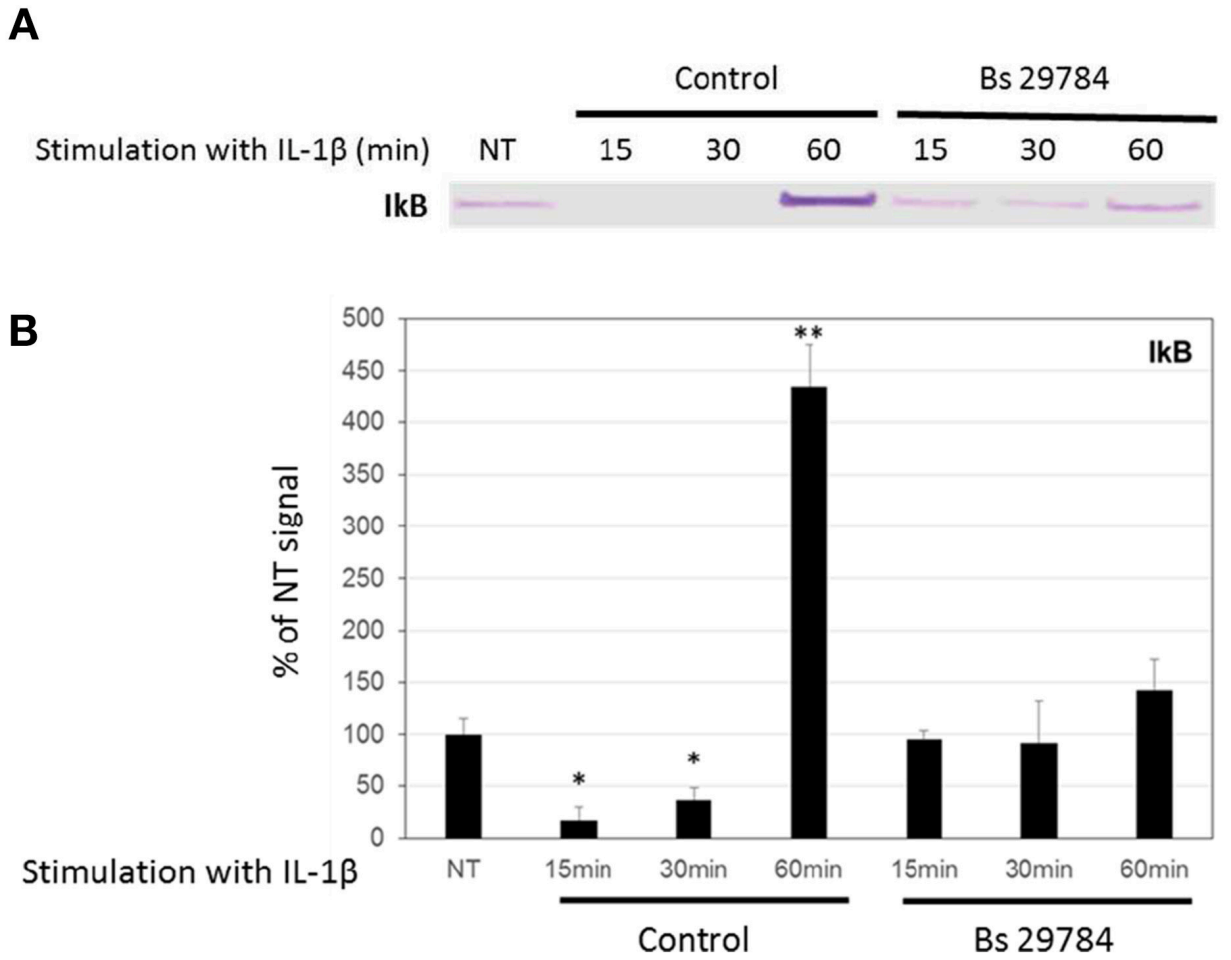
Bs 29784 prevents the breakdown of the NF-κB inhibitor, IκBα. Caco-2 cells were pretreated apically with Bs 29784 for 16 h prior to basolateral stimulation with IL-1β (20 ng/ml) for 0, 15, 30, or 60 min. Cytolosic levels of IκBα were visualized by Western-blot **(A)**. Band densities were measured using Image J software and subjected to statistical analysis **(B)**. ^*^ and ^**^Significant differences between the groups, *p* < 0.05 and *p* < 0.01, respectively.

Nuclear translocation of NF-κB/p65 is prevented by binding to cytosolic IκBα, and its release requires proteasome-mediated degradation of IκBα. Figures 5A,B shows that IL-1β treatment of Caco-2 cells caused a rapid and significant (*p* < 0.01 and 0.05) degradation of IκBα after 15 and 30 min (17.2 and 36.7% of control signal, respectively) but a rebound at 60 min, due to the fact that IκBα expression itself is NF-κB dependent (35). Interestingly, pretreatment of Caco-2 cells with Bs 29784 prevented IL-1β from causing significant IκBα degradation [94.9, 92.2, and 142.1%, (*p* < 0.05), of control signal, after 15, 30, and 60 min of IL-1β exposure, respectively] suggesting that Bs 29784 has a positive effect on the modulation of the inflammatory response by inhibiting the degradation of IκBα.

### Protective Effect of Bs 29784 Involve Secreted and Cell-Associated Factor(s)

**Figure 6:**
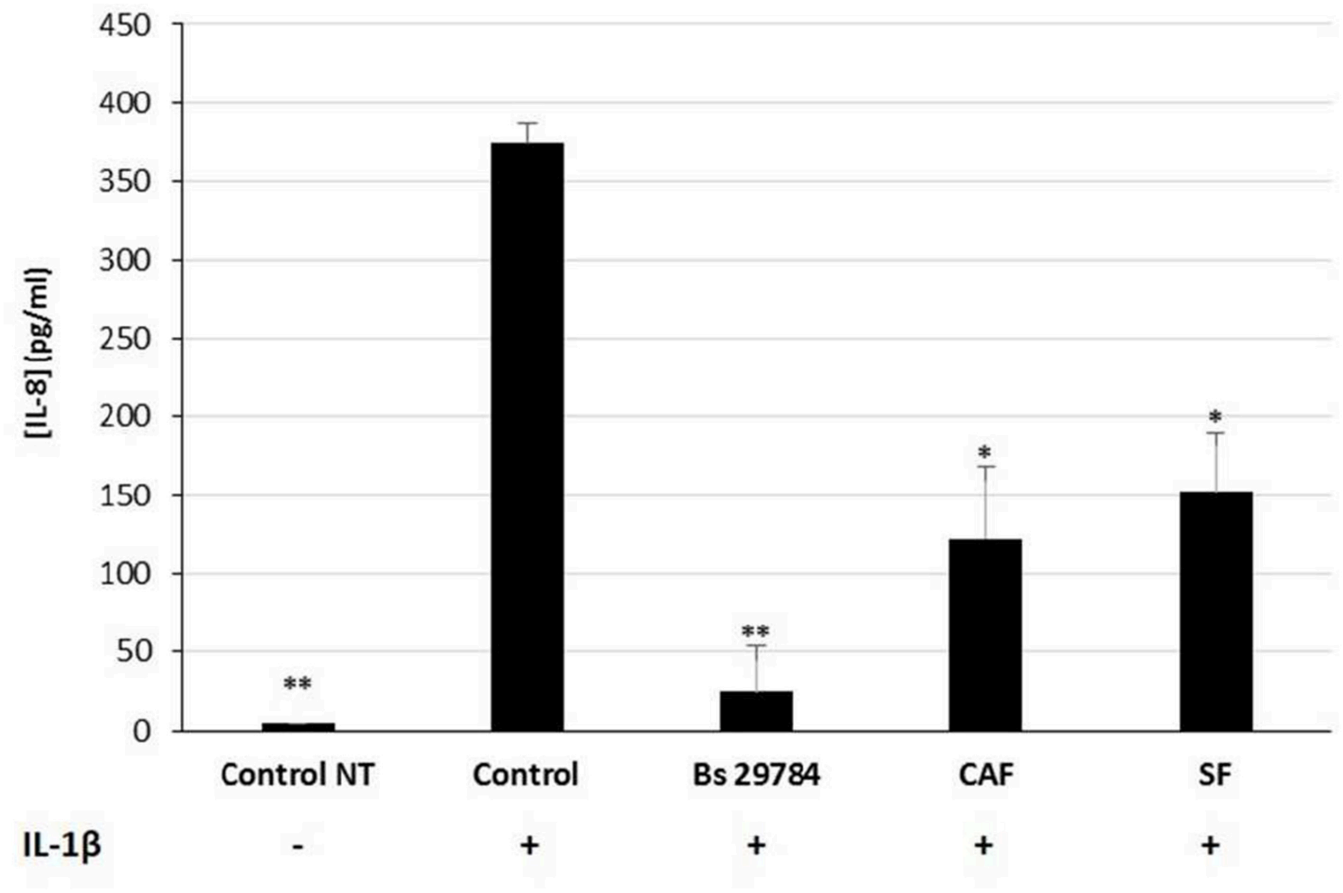
Contribution of soluble and membrane-bound factors to anti-inflammatory effects of Bs 29784. Caco-2 cells were pretreated apically either with live secreting cells (Bs 29784), with PFA-killed Bs 29784 (Cell Associated Factors, CAF) or with Bs 29784 physically separated from the Caco-2 cells by an additional filter (Secreted Factors, SF). After 16 h, the cells were treated basolaterally with IL-1β (20 ng/ml) and IL-8 released in the next 6 h was measured by ELISA. ^*^ and ^**^Significant differences between the groups, *p* < 0.05 and *p* < 0.01, respectively.

We next evaluated the nature of the bacterial factors that exert the observed immunomodulatory effects by Bs 29784 in IL-1β-stimulated Caco-2 cells. Caco-2 cells were thus exposed to Bs 29784 secreted factors (SF) or to PFA-killed Bs 29784 (also noted as cell-associated factors, CAF). As shown in Figure 6, both SF and CAF, were able to reduce IL-8 secretion upon stimulation with IL-1β (59.2 and 67.3% of inhibition (*p* < 0.01), respectively, compared to IL-1β-treated control). However, compared to live Bs 29784 causing 93.3% of inhibition (*p* < 0.001), their protective effect was reduced. This indicates that both Bs 29784 cells and its secreted compounds are involved in the control of IL-8 production.

### Bs 29784 Reduces Induction of iNOS by Pro-inflammatory Cytokines

**Figure 7:**
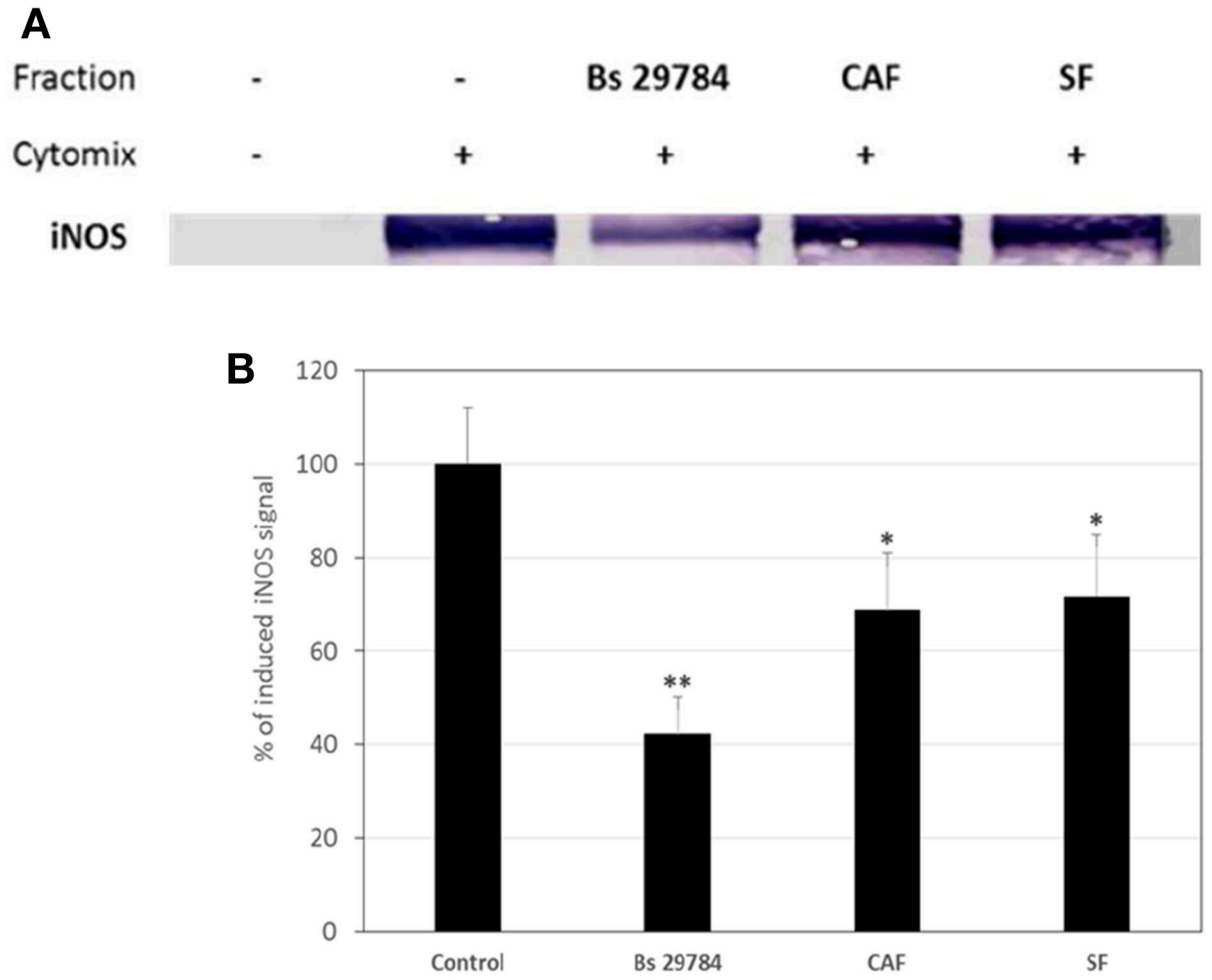
Blunted cytokine-induced upregulation of iNOS due to pretreatment of cells with Bs 29784. Caco-2 cells were pretreated with Bs 29784: live secreting cells (Bs 29784), PFA-killed cells (CAF), or secreted factors from BS 29784 (SF), or not, and subsequently stimulated basolaterally with Cytomix for 24 h. Cytolosic levels of iNOS were visualized by Western-blot **(A)**. Band densities were measured using Image J software and subjected to statistical analysis **(B)**. ^*^ and ^**^Significant differences between the groups, *p* < 0.05 and *p* < 0.01, respectively.

Thereafter, we evaluated if Bs 29784 was able to limit the NF-κB dependent induction of iNOS in simulated inflammatory conditions. Caco-2 cells pre-treated with or without Bs 29784 were exposed to a mixture of pro-inflammatory cytokines (IFNγ, TNFα, and IL-1β) called Cytomix before analysis of iNOS expression by Western-blot. Results (Figure 7) demonstrated that live Bs 29784 bacteria were able to inhibit Cytomix-mediated induction of iNOS (57.7% of inhibition compared to cytomix-treated cells, *p* < 0.01). SF and CAF were also tested and displayed moderate activity compared to live bacteria [28.3 and 31.2% of inhibition compared to cytomix-treated cells, for secreted factor(s) and PFA-killed bacteria, respectively, *p* < 0.05], indicating that here also, Bs 29784 cells and its secreted compounds are involved in regulation of iNOS induction.

## Discussion

The intestine absorbs food with great efficiency, and is also armed with a large amount of immune cells ready to intervene when micro-organisms from the large local microbiome breach the intestinal epithelium. In fact, intestinal epithelial barrier is one of the major defense mechanisms against invasion. Nutritional or pharmacological interventions aimed at reinforcing this barrier are being actively sought after for human and animal health.

In the livestock industry, where the prophylactic and growth-promoting use of antibiotics is being restricted in more and more countries, an increasing number of ingredients is being developed to protect digestive health and prevent increased leakiness of the gut and increased intestinal inflammation (39). Probiotics, also referred to as Direct-Fed Micro-organisms, are an attractive group of ingredients, and some have shown promising effects on gut barrier function, associated gains in health, such as the control of the inflammatory response, and productivity. *Bacillus*-based probiotics are especially popular due to the fact that they can be formulated as stable spores and can be therefore be used in processed feed. However, the mode of action of many *Bacillus* species and strains used as feed additive is not well-understood.

In the present study, we first tested how three *B. subtilis* strains affected intestinal-epithelial integrity. Surprisingly, we found that only Bs 29784 actually strengthened barrier function as reflected by increased trans-epithelial potential differences (Figure 1). In contrast to Bs 29784, Bs B had no effect, and Bs A weakened epithelial resistance, indicating a deleterious effect on barrier integrity. The effect of Bs 29784 on TEER was associated to an increase in the expression of the main proteins involved in tight junctions, i.e., ZO-1, occluding, and claudin-1 (Figure 2) in accordance with previously published results obtained with the porcine intestinal cells IPEC-J2 (40).

One reason of focusing on intestinal epithelial cells is that probiotics likely mainly interact directly with these cells, and another is that these cells are increasingly being recognized as regulators of inflammation beyond their absorptive role. Indeed, Intestinal Epithelial Cells (IEC) secrete a variety of cytokines and chemokines (41), which orchestrate immune responses and regulate the recruitment of immune cells. One such chemokine is IL-8, which mainly attracts neutrophils and thus, increases local inflammatory response. Since the *B. subtilis* strains differentially affected TEER we next explored their possible immunomodulatory effects and tested if and how they are able to regulate inflammatory processes in Caco-2 cells. Our results showed again that the effect of *Bacillus* on IL-8 secretion by IEC is rather strain-specific.

When we tested the immunomodulatory effects of the three *Bacillus* strains, we first noticed that strains themselves slightly stimulated IL-8 release by IEC (Figure 3) which is in line with previous studies reporting a mild stimulation of IL-8 release from Caco-2 cells with *Bacillus* strains probiotic (42). Nevertheless, the magnitude of IL-8 secretion was different between the three strains, with Bs 29784 being the weakest stimulator. When cells pre-treated with *B. subtilis* were subsequently challenged with different, potent pro-inflammatory molecules, it was observed that the IEC produced significantly less IL-8, suggesting that the treatment with bacterial strains had programmed the cell to cause a milder inflammatory response. One benefit of this attenuating effect could be that these strains could be useful in limiting inflammatory responses during intestinal injury or when intestinal macrophages fail to downregulate IL-1β production during chronic intestinal inflammation (43).

Interestingly, Bs 29784 exhibited a stronger anti-inflammatory effect than the two other strains tested. Bs 29784 also exerted its immunomodulatory property in all stressed conditions used here, whereas Bs A and Bs B strains potentiated the pro-inflammatory effect of DON and Bs A also potentiated the pro-inflammatory effect of flagellin.

The mechanistics behind these discrepant effects are not clear. Of all three *B. subtilis* strains tested, it thus seemed that Bs 29784 was the most potent modulator of inflammatory responses. To gain insight into the mechanism, we tested, in IL-1β stimulated conditions, whether Bs 29784 limits the translocation of NF-κB from the cytosol into the nucleus. Indeed, while initially stimulating nuclear translocation of NF-κB to a small extent (in agreement with the observed slight stimulation of IL-8 release), Bs 29784 strongly inhibited the IL-1β dependent translocation of NF-κB into the nucleus (Figure 4). Conversely, IκB, a molecule that inhibits NF-κB translocation, did not decrease in Bs 29784 pre-treated Caco-2 cells after IL-1β challenge (Figure 5).

Previous studies demonstrated an immunomodulatory effect of bacterial soluble compounds, some of which act by inhibiting the NF-κB signaling pathway, or of bacterial membrane-associated compounds, but rarely both (44–48). Interestingly, the protective effect of Bs 29784 can likely be attributed to the cells themselves and to the secreted compounds since, both of these were able to exert an anti-inflammatory effect that was lower than that of the combination (Figure 6).

The use of Caco-2 cells may, in some respects, seem too remote from the context in which the Bs 29784 strain is used. However, in the absence of a clearly established avian *in vitro* model, it constitutes a first step that allowed us to demonstrate the direct immunomodulatory potential of Bs 29784. Beyond this direct effect shown in this study, we must also consider the indirect effect of Bs 29784, by acting on the microbiota through the compounds it secretes. Identifying them would shed light on the mode of action of this probiotic strain which we already have shown to have an impact on the animal microbiota (49).

Our results indicate that *B. subtilis*-based probiotics help control the release of IL-8 by IEC in response to several pro-inflammatory stimuli. We did not measure other cytokines, but obtained additional evidence of an anti-inflammatory effect, namely the reduction of NF-κB dependent iNOS upregulation upon exposure to pro-inflammatory cytokines (Figure 7) likely due to the sequestration of NF-κB in the cytosol. iNOS is able to produce large amounts of NO, which helps destruct pathogens, but may be harmful if uncontrolled (33, 34). Therefore, iNOS expression should not be completely inhibited but tightly regulated, as Bs 29784 seems to do, to prevent excessive inflammation.

Taken together, our results show that *B. subtilis*-based probiotics do possess properties that may help attenuate and prevent inflammatory responses in the intestine while also strengthening the gut barrier; a key property that helps prevent potentially sustaining chronic inflammation. In this respect, we speculate that the growth-promoting effect of Bs 29784, previously shown in poultry (23), could be explained in part by an immune modulating action created by a combination of metabolites and membrane-bound factors. Similar protective effects of *B. subtilis* strains were already observed, in other systems, including *in vivo* models (50–53). However, the results also show that not all *B. subtilis* strains supports these effects equally and that important differences may exist in the efficacy which these promising feed and food additives may act positively in inflammatory challenged conditions and promote gut health.

## Data Availability

The datasets generated for this study are available on request to the corresponding author.

## Author Contributions

LR, ED, and MM designed the experimental strategy. MM, CN, and JP performed the experiments. MM and LR analyzed the experimental data. MM, EE, and LR wrote the manuscript. All authors revised and approved the final manuscript.

### Conflict of Interest Statement

LR, EE, and ED are employed by Adisseo France SAS. KB and SC are employed by Novozymes A/S. Adisseo France SAS and Novozymes S/A jointly commercialize Bacillus strain Bs 29784. The remaining authors declare that the research was conducted in the absence of any commercial or financial relationships that could be construed as a potential conflict of interest.
